# Influence of granite micro particles on the mechanical, damping and antimicrobial properties of silk–sisal hybrid composites

**DOI:** 10.1038/s41598-024-63190-w

**Published:** 2024-07-10

**Authors:** K. Sripriyan, M. Karthigha

**Affiliations:** 1 School of Mechanical Engineering, VIT Bhopal University, Bhopal-Indore Highway, Kotrikalan, Sehore, Madhya Pradesh 466 114 India; 2 Department of Computer Science and Engineering, PSG Institute of Technology and Applied Research, Neelambur, Coimbatore, 641 062 India

**Keywords:** Silk–sisal plain weave fiber mat, Granite-micro-filler, Mechanical properties, Fracture morphology, Damping characteristic, Physical properties, Engineering, Materials science

## Abstract

This paper focuses on the effect of granite-micro-filler on static mechanical properties, damping behavior, and physical characteristics of silk-sisal hybrid composite. The composites structures are fabricated by using three layers of plain weaves mat with varying weight percentages (2, 4, and 6 wt%) of granite-micro-filler by hand layer process. The effect of granite-micro-filler on silk-sisal is analyzed and results are compared with pure silk-sisal (without granite-Micro-filler) hybrid composite. The results infer that; the tensile strength and flexural strength value of the developed silk-sisal hybrid composites are increased with the increase in wt% of granite-micro-filler content than pure silk–sisal, approximately 5% and 9% improved value in order by adding 6 wt% granite-micro-filler on silk–sisal. The tensile and flexural fracture morphology analysis indicated that composed plain weave of silk–sisal fiber mat with two (longitudinal and transverse) directions possesses higher mechanical properties and also observed that, granite-micro-filler dispersed on silk–sisal fiber mat and resin with closely packed. The improved damping factor is obtained by adding 6 wt% of granite-micro-filler content, which is approximately 75.8% higher than pure silk–sisal. The physical properties of silk–sisal hybrid composite have also been analyzed and well discussed in this paper.

## Introduction

In the past few decades, natural fiber composites are one the better alternative for many industrial and householding applications, based on their properties viz., low cost, specific strength, good thermal and acoustic, and biodegradability^1^. Many researchers have tried natural fibers such as sisal, banana, jute, flax, coir hemp, palmyra, kenaf, etc. for various engineering applications^2^, due to low load application, etc.^3^.

However, composite material performance was generally addressed by improvement of tensile strength, flexural strength, hardness, compression, and impact strength of their compositions. Further, physical and damping properties were also studied for the specific application extensively^4^ and^5^. The above characteristics were enough to identify the material performance under various engineering applications^6^. Further improvements may lead to addressing innovative new applications, in this connection, a new attempted has been made in this study. The existing outcome of sisal- banana hybrid composite was replaced by silk–sisal weave mat hybrid composite added with a different weight percentage of granite-micro-fillers to improve the dynamic characteristics and possess the mechanical properties. In general, may natural fibers were used as reinforcing materials for fabricating hybrid composite, owing to (i) low cost, (ii) light weight, (iii) high specific strength (iv) high damping characteristics(v) biodegradable nature^7^ and^8^. Along with that, fiber orientation in plain weaving with different layers also be give more response^9^ and^10^, i.e. random orientation may give higher Young’s modulus, whereas higher loading may in the transverse direction^11^.

In the year of 2014^3^, studied the free damping behavior of short sisal-banana fibers. They were tried with different fiber lengths (30, 40, and 50) and weight percentages (2, 4, 6, 8, and 10) and concluded as an increase in fiber content in wt% increases the tensile, flexural, and impact and also a notable improvement in damping behavior. In the same year^12^ investigated the effect of redmud on banana polyester composite. The researcher was studied in different partial sizes with different wt%. The mechanical and free damping results inferred that 50% of improved mechanical properties were observed and 4 lm of redmud and 8 wt% of filler had more influence.

Graphene-basalt Micro-filler on the banana-sisal composite structure was studied to address the mechanical and vibration behavior for various engineering applications^13^.

The research outcome was graphene addition gives 1.5 times higher flexural strength than basalt. In the year 2019^14^ a silk composite was developed with flax fiber, and the performance was analyzed by conduction mechanical and interfacial tests. The results inferred that flax has a significate effect to enhance the impact energy whereas silk prevents crack propagation.

A glass fiber laminated composite was developed with a banana. Sisal and E-glass fibers as reinforced with epoxy. Nine different compositions of two and three layers structures were developed, and the results revealed that two and three layers composites improve the tensile strength and banana–sisal with two layers observed as higher flexural strength^15^. Silk reinforced gelatin composites were developed by Quazi T.H. Shubhra and weathering test was conducted to understand the environmental effect^16^. Approximately 52 percent of weight loss was observed in the biodegradation test within 24 h.

A water absorption test was performed on jute and carbon epoxy composites for that six layers of jute and four layers with different stacking sequences followed. The outcome of the studies was 3.8 percent of water absorption observed and also they emphasized the different fiber orientations be significate importance in water absorption^17,18^. In the extended literature survey on various fibers, banana-sisal possesses higher mechanical and physical properties. To replace and improve the damping behavior, the silk–sisal plane weave fiber was proposed in this study by adding different weight percentages of granite-Micro-filler. The silk–sisal hybrid composite was fabricated with three layers of bidirectional orientation by hand lay-up methods. The developed silk–sisal composite was examined by tensile, flexural, and impact analysis, and also free vibration and physical properties were studied and the results are compared with the pure silk–sisal hybrid composite.

In this paper is to reveal the impact of bidirectional silk–sisal hybrid composite structure and its potential to enhance the mechanical, damping, and physical behavior under varying granite-micro-particles at 2 wt%, 4 wt%, and 6 wt%, as filler materials. Further, relationship between their mechanical and damping characteristics was observed to investigate the failure behavior due to granite-micro-particles relative wt% and to develop a lightweight structures for automotive component, construction industries and machine support industries applications.

## Materials and preparation

### Materials involved

In this study, silk–sisal are used as the reinforcement material with different weight percentage of filler of granite Micro-powder. Epoxy-based resin (LY556 grade) and hardener (HY951 grade) are used as matrix and 10:1 blending ratio of the resin with the hardener is proposed in this study. Granite powder (106–124 μm) is used as the Micro-filler for the improvement of damping properties. All the materials are purchased from local resources and the results of the proposed silk–sisal hybrid composite are compared with existing results of sisal-banana composition, properties of the hybrid composite of silk, banana, and sisal fibers are given in Table 1^19^,^20^.

**Table 1:** Properties of silk, banana and sisal fibers.

S.No	Name of the composite	weight %	References	Tensile strength in (MPa)	Flexural strength (MPa)	Impact strength (J)	Damping
1	Banana	No filler	4, 7	15.60	68.80	1.60	0.038
2	Sisal		6, 23	24.50	80.45	1.76	0.034
3	Sisal & banana		3, 5, 28	18.50	57.47	1.71	0.035
4	Silk		2, 23, 29	19.20	51.96	1.25	0.33

Initially, the fibers are cleaned with normal water in several times and allowed to dry at room temperature for the day. The NaOH solution is used to remove the excess debris from the fibers, and soaked in the solution for one day. Finally, washing is carried out with purified water and then allow to dry in a hot oven at 70 degrees Celsius for 3–4 h. The fibers are sliced into a span of 300 mm for moulding the composites.

### Granite micro-filler

In recent investigation inferred that granite composite material is one of the substitutes for cast iron for machine tool structures. Which is possess higher specific stiffness, damping ratio, thermal stability, etc. In this study, granite powder of 106–124 μm is selected as the Micro-filler to improve the damping properties of the silk–sisal hybrid composite.

### Epoxy and hardener

The selection of a proper binder plays a vibrant role as it provides strength to the mixture used. From the literature study, epoxy resin is selected due to its ability to adjust to the physical and chemical changes of the structure^21^. In this study Epoxy LY556 (density 1150–1390 kg/m^3^), mixed with hardener HY951 (density 985–998 kg/m^3^) is used to prepare the composite structure^9^.

### Composite preparation

**Figure 1:**
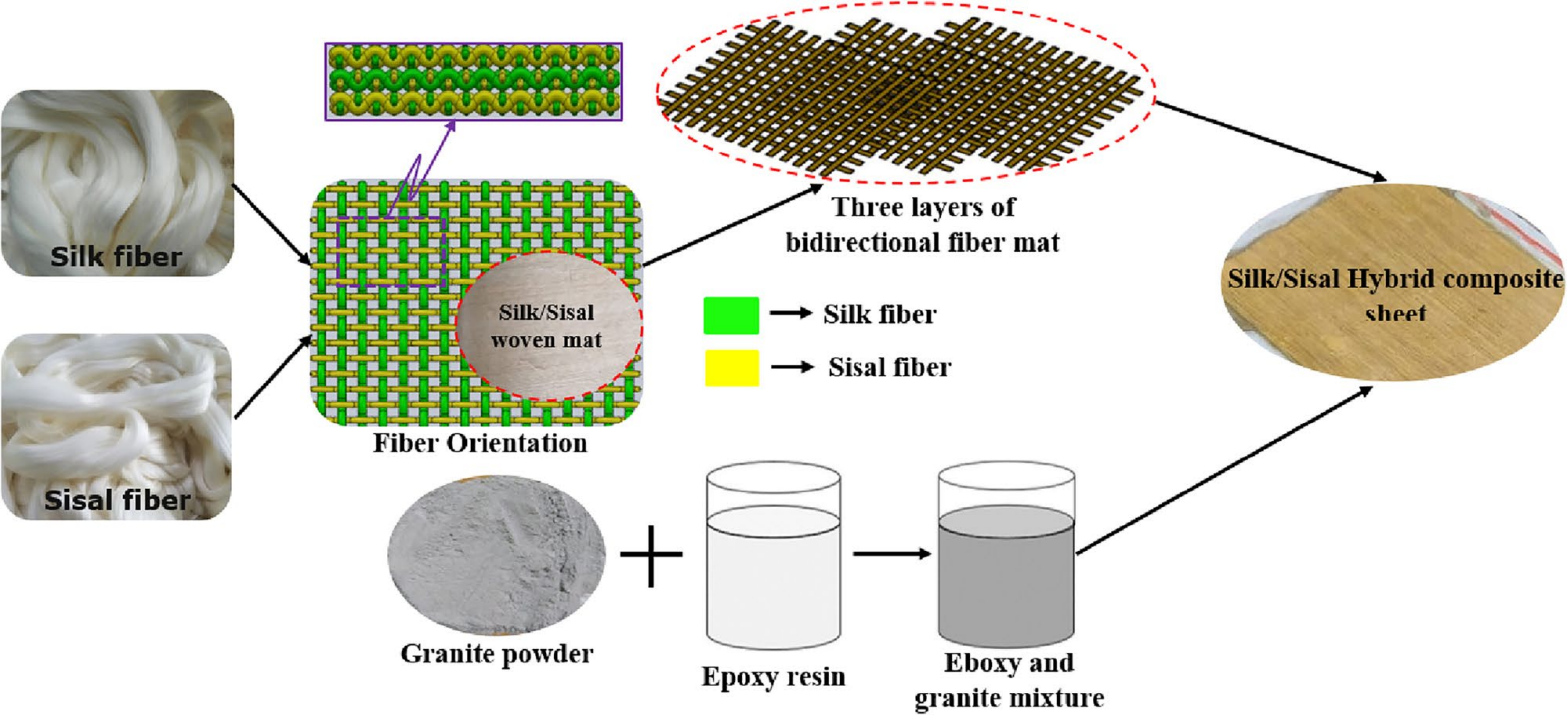
Typical view of steps involved to prepare the granite-micro-filler on silk–sisal hybrid campsite structure.

The manual hand layup open molding technique is adopted to prepare the composite (Three-layer bidirectional fiber mat) structure. At first, the eligible sunlight-dried silk–sisal fibers are separated and chopped with a maximum length of 300 mm. Then weft yarns are woven across (above and below) the warp yarns. In this fiber weaving process, the warp is considered silk fiber, and the weft is considered sisal fiber as shown in Fig. 1. The composite is performed by arranging three layers of silk–sisal fibers mat unidirectional. For that, mould was made with rubber surround of 300 × 300 × 3 mm dimension. Granite-micro-filler (106 μm—124 μm) was used as a Micro-filler in the range of 2 wt%, 4 wt%, and 6 wt%. which was mixed with epoxy resin (LY536) and hardener (HY951) by hand stirring until all the Micro-fillers are evenly dispersed. Four different hybrid composite structures are prepared for different Micro-filler ratios of 2 wt% granite with silk–sisal (sample 1), 4 wt% granite with silk–sisal (sample 2), 6 wt% granite with silk–sisal (sample 3), and 0 wt% granite with silk–sisal (sample 4) i.e. without filler.

Silk–sisal fiber bidirectional mats have been located layer-up in 90-degree orientation by a cross-lay pattern. The Epoxy resin was mixed with the granite-micro-filler and it was applied over all the layers of the reinforcement. Subsequently, rollers are used by hand to roll over the wet composite to ensure an enhanced interaction between the reinforcement and the matrix, facilitate a uniform resin distribution, and obtain the required thickness of 3 mm. Finally, the hybrid composites are left to cure under standard atmospheric conditions. Steps involved to prepare the granite-micro-filler on silk–sisal hybrid campsite structures are shown in Fig. 1.

### Ethical approval

The authors state that the research work was conducted according to ethical standards.

## Results and discussion

The hybrid granite-micro-filler on silk–sisal composite structure (300 mm × 300 mm) was developed by hand layup technique. There are three sets of four different compositions of hybrid composite structures made. All the samples are prepared according to the ASTM standard for testing of mechanical, viz, Tensile Strength, Flexural Strength, and Impact Strength, to understand the significance of granite-micro-filler on the silk–sisal hybrid structure. In addition, the rate of water absorption and degradation characteristics were discussed to explore for selection of one-time usable biodegradable materials. Finally, a morphological study was performed to understand the fracture behavior and pullout of the developed composite structure.

### Significance of silk–sisal on mechanical properties

#### Tensile strength

**Figure 2:**
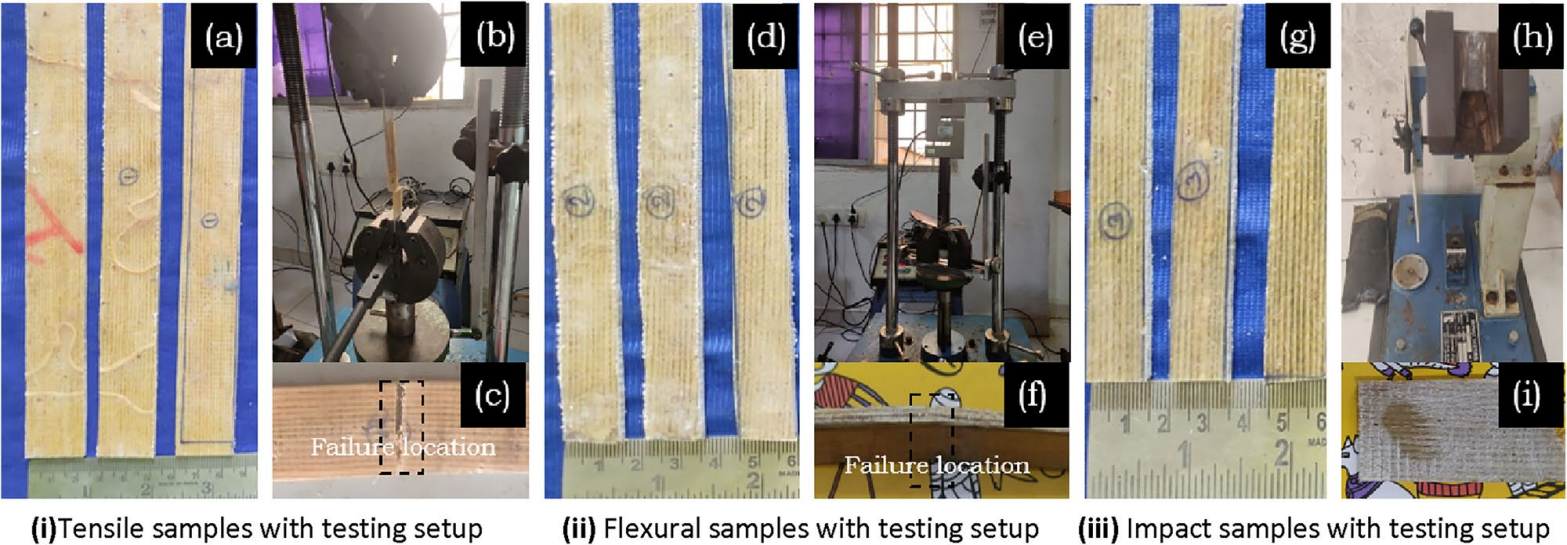
Typical unprepared developed silk–sisal hybrid testing samples with the test setup.

Tensile test samples are carried out to understand the significance of granite-micro-filler on tensile strength by ASTM: D3039^13^ and^22^. The loading speed is maintained at 5 mm/min for each test, each typical unprepared samples are shown in Fig. 2a–i. The tensile strength values are calculated by conducting three samples in each composition and the average Tensile values of pure (Silk + sisal) and hybrid composites (Silk + sisal + granite filler) were reported in Table 2.

**Table 2:** Properties of developed hybrid composite.

S. No	Name of the composite	Weight %	No of samples	Tensile strength in (MPa)	Flexural strength (MPa)	Impact strength (J)	Damping
1	Silk–sisal reinforced composite	No filler	1	22.60	84.60	0.90	0.37
			2	24.60	91.90	0.50	0.31
			3	23.30	97.60	0.80	0.37
			Average	24.00	91.37	0.733	0.033
2		2 wt% of filler	1	20.60	117.90	0.70	0.04
			2	19.9	102.80	0.80	0.03
			3	23.70	81.64	0.90	0.03
			Average	21.00	100.78	0.80	0.036
3		4 wt% of filler	1	27.10	114.90	0.60	0.06
			2	21.00	99.78	0.80	0.03
			3	23.50	123.90	0.70	0.03
			Average	24.00	112.86	0.70	0.043
4		6 wt% of filler	1	28.60	101.5	0.60	0.03
			2	19.30	99.25	0.90	0.03
			3	27.70	97.28	0.70	0.40
			Average	25.20	99.34	0.733	0.058

**Figure 3:**
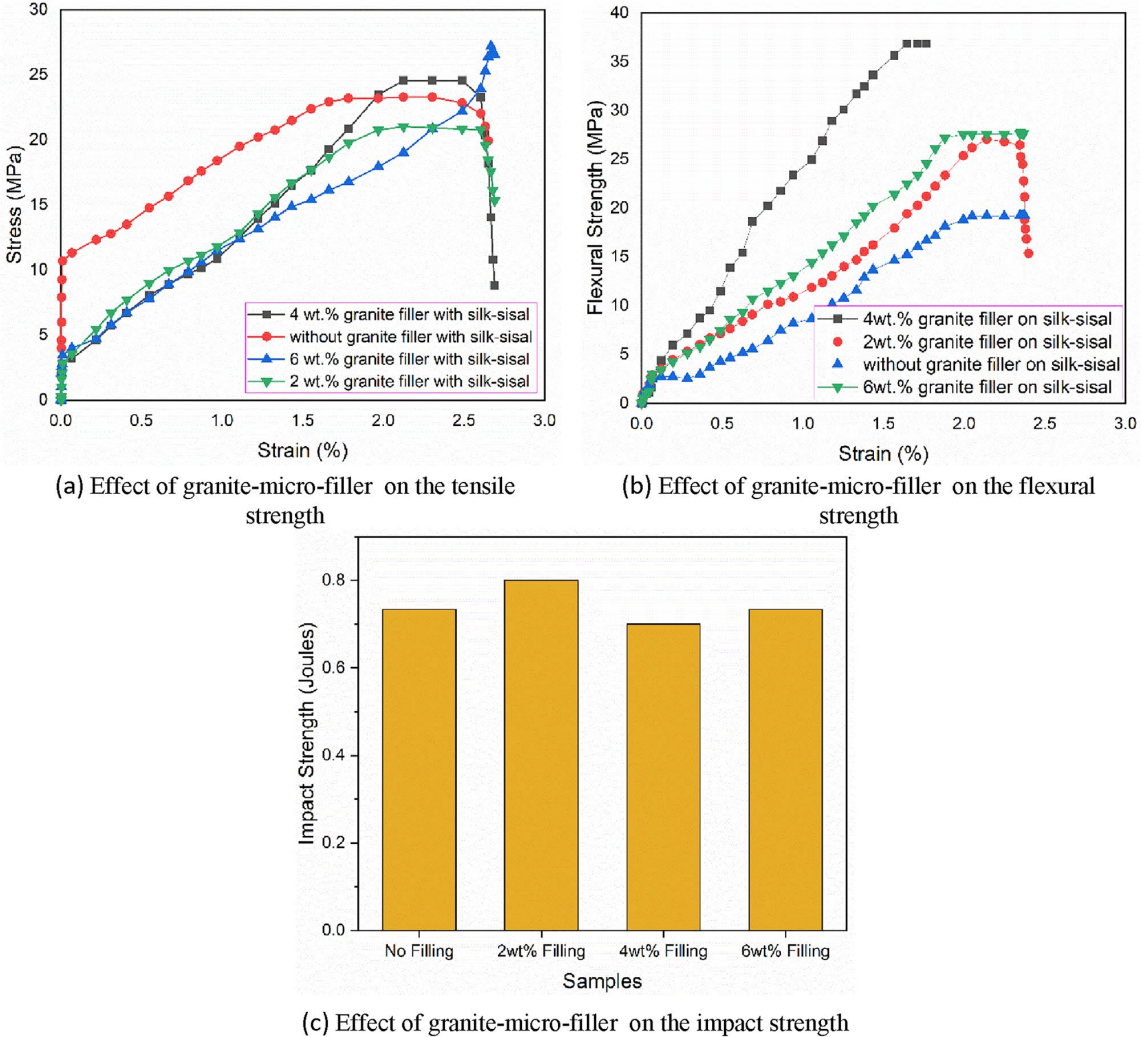
Stress vs Strain curve.

The average value of pure silk–sisal is 23.4 MPa whereas 21 MPa, 24 MPa, and 25.2 MPa were obtained respectively for granite-micro-filler weight percentages of 2, 4, and 6 on silk–sisal. The comparative responsible plot of the stress–strain curve for all four samples is shown in Fig. 3a. There were notable improvements in the tensile strength value by adding granite-micro-filler to the silk–sisal hybrid composite. This is due to proper dispersion of granite-micro-filler and matrix/fiber/Micro-filler interface bonding makes a tendency to possess more tensile force (Wu et al., 2019) and^23^. These results evidenced that, the silk–sisal hybrid composite combination without Micro-filler itself possesses better tensile strength value due to its nature than banana-sisal fibers composition, which is approximately 20.85% higher, as detailed in Tables 1 and 2.

Further Fig. 3a infers that the increasing wt% of granite-micro-filler is found for increasing tensile strength value, except for 4 wt%. It directly implies that uniform dispersed wt% of granite-micro-filler on silk–sisal hybrid composites will pose a higher tensile strength value. However, among differences in wt% of granite-micro-filler on silk–sisal hybrid composites can have better strength compared to without filler on silk–sisal. But many researchers have pointed out, that increasing content of Micro-filler may lead to occur failure due to its development of agglomeration, and also the stress concentration at the agglomeration of Micro-filler may cause unexpected failure to the fabricated hybrid composite structure^12^.

The present results inferred that 1.05 times increased tensile strength value showed a 6 wt% of granite-micro-filler content than pure silk–sisal (without Micro-filler). Similarly, 0.8 times higher and no acceptable changes are observed in 2 wt% and 4 wt% of granite-micro-filler content respectively. Finally, 6 wt% of granite-micro-filler content is found to be a more significant factor in desired tensile strength. Further, the variation of tensile strength value of different Micro-filler content is discussed with the aid of a fractography image under section "Microstructure and fracture morphology".

#### Flexural strength

Flexural strength is one of the testing methods to calibrate the rupture behavior of the composite structure^15^. The granite-micro-filler on silk–sisal hybrid samples was prepared according to ASTM: D790, details in Fig. 3d–f, and maximum bending load is applied at a rate of 5 mm/min till the samples get fractured/broke. Testing was conducted for three samples in each composition and the average flexural values of pure (Silk + sisal) and hybrid composites (Silk + sisal + granite filler) were reported in Table 2.

The variances in flexural strength of silk–sisal with the effect of wt% of granite-micro-filler content are studied and the average value of pure silk–sisal is 91.37 MPa whereas 100.78 MPa, 112.86 MPa, and 90.63 MPa found respectively for granite-micro-filler weight percentages of 2, 4, and 6 on silk–sisal. The relatively responsible plot of the stress–strain curve for all the four compositions is shown in Fig. 3b. It was noted that increasing the weight percentage of granite-micro-filler is representing bell-shaped trends rather than increasing flexural strength. However, a drastic change in flexural strength is observed by adding from 2 to 4 wt% of granite-Micro-filler, which is around 10.7% higher whereas a sudden drop of around 19.6% by changing from 4 to 6 wt%. This is due to the reason non-uniform dispersed wt% of granite-micro-filler on silk–sisal hybrid composites^24^. The experimental results evident that silk–sisal may be an alternative hybrid composite of banana-sisal to enhance the flexural strength, even without adding granite-Micro-filler.

The present results revealed, 1.25 times higher flexural strength value exposed at 4 wt% content of granite-micro-filler than pure silk–sisal (without Micro-filler). Similarly, 1.1 times higher and no change in flexural strength values are obtained for 2 wt% and 6 wt% of granite-micro-filler content respectively. Finally, 4 wt% of granite-micro-filler content is found to be a significant factor in desired flexural strength. Further, the variation of flexural strength value of different Micro-filler content was discussed with the aid of a fractography image under section "Microstructure and fracture morphology".

#### Impact strength

The impact competence of the unnotched hybrid composite under the unexpected applied load is studied by ASTM: D481 for four compositions of samples, i.e. kinetic energy applied to the hybrid composite samples to initiate the fracture and to continue until the breakage^16^. Typical samples are shown in Fig. 2g–i and the average impact strength values of pure (Silk + sisal) and hybrid composites (Silk + sisal + granite filler) were tabulated in Table 2.

The altered impact strength is observed for different wt% of granite-micro-filler content. This may be the reason for non-uniform dispersion at the fiber mat. The average impact strength value of pure silk–sisal was 0.75 J whereas 0.83 J, 0.6 J, and 0.6 J were found for granite-micro-filler wt% 2, 4, and 6 on silk–sisal respectively. The relatively responsible plots for all four compositions are shown in Fig. 3c.

However, a severe variation in impact strength is observed by adding granite-Micro-filler, which is around 27.7% lesser by adding 2–4 wt% whereas no change by adding from 4 to 6 wt%. On the other hand, there is an improvement in fibers mat and matrix (resin + granite-Micro-filler) bond, which led to increases in the tensile and flexural strength as detailed in Fig. 3a and b.

The impact results indicated a 1.1 times higher impact strength value explored at 2 wt% content of granite-micro-filler than pure silk–sisal (without Micro-filler). Similarly, 0.99 times lower and the same impact strength values are observed for 2 wt% and 6 wt% of granite-micro-filler content. The fracture study is conducted to reveal the presence of granite-micro-filler on silk–sisal hybrid composite.

#### Damping factor

**Figure 4:**
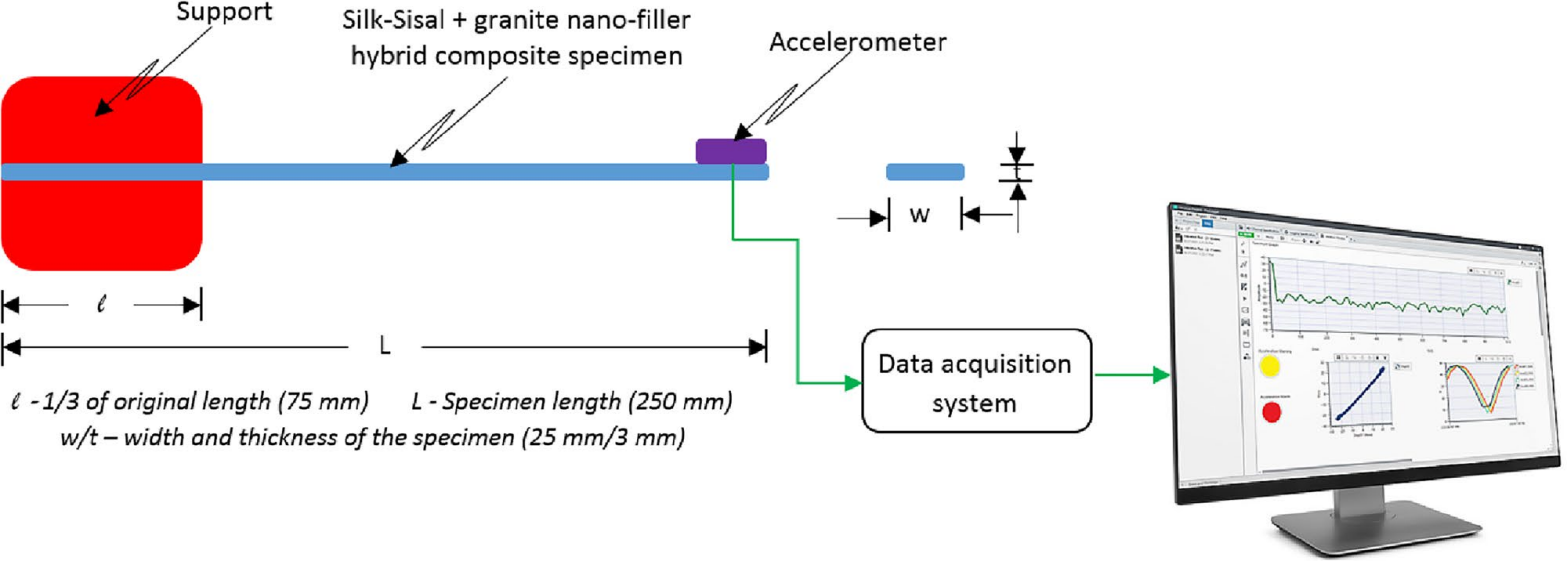
Schematic view of fee vibration setup.

The dynamic characteristics of developed hybrid composite structures (pure and different granite-micro-filler content) are studied under the free transverse vibration^20^ and (Sripriyan K, Prashanth M, Ramu M, 2017). According to the literature survey for the free vibration test, cantilever samples are prepared as per ASTM: E756 standard of 250 × 25 × 3 mm^25^. The line diagram of the experimental setup was shown in Fig. 4, which comprises the accelerometer, DAQ (Data Acquisition System), and display unit (ME ‘scope). The free vibration responses were uninterruptedly monitored and recorded using an accelerometer attached at the free end of the hybrid composite structure and damping ration 'ζ' (Zeta) calculated based on the logarithmic decrement to experimental systems^11^. The motion takes from Eq. (1).

1 $$x\left(t\right)=X {e}^{-\upzeta {\omega }_{n}\text{t}}\text{sin}({\omega }_{d}t-\varphi )$$

The above equation is indicted that the hybrid composite structure oscillates with a frequency ${\omega }_{d}=(\sqrt{1-}\upzeta$)${\omega }_{n}$. As ζ (zeta) is less than 1, ${\omega }_{d}$ is always less than ${\omega }_{n}$. Where ζ is the damping factor to be calculated, ω_n_ is the natural frequency of the developed hybrid composite structure, X and $\varphi$ are the amplitude and phase angle response at initial conditions, and n is the ratio of two successive oscillations. The free vibration can be expressed in Eq. (1). and the actual damping ratio can be found in Eq. (2).

2 $$\delta =\frac{{x}_{n}}{{x}_{n+1}}=\text{exp}\left(\frac{2n\upzeta \pi }{\sqrt{1-{\upzeta }^{2}}}\right)$$

**Figure 5:**
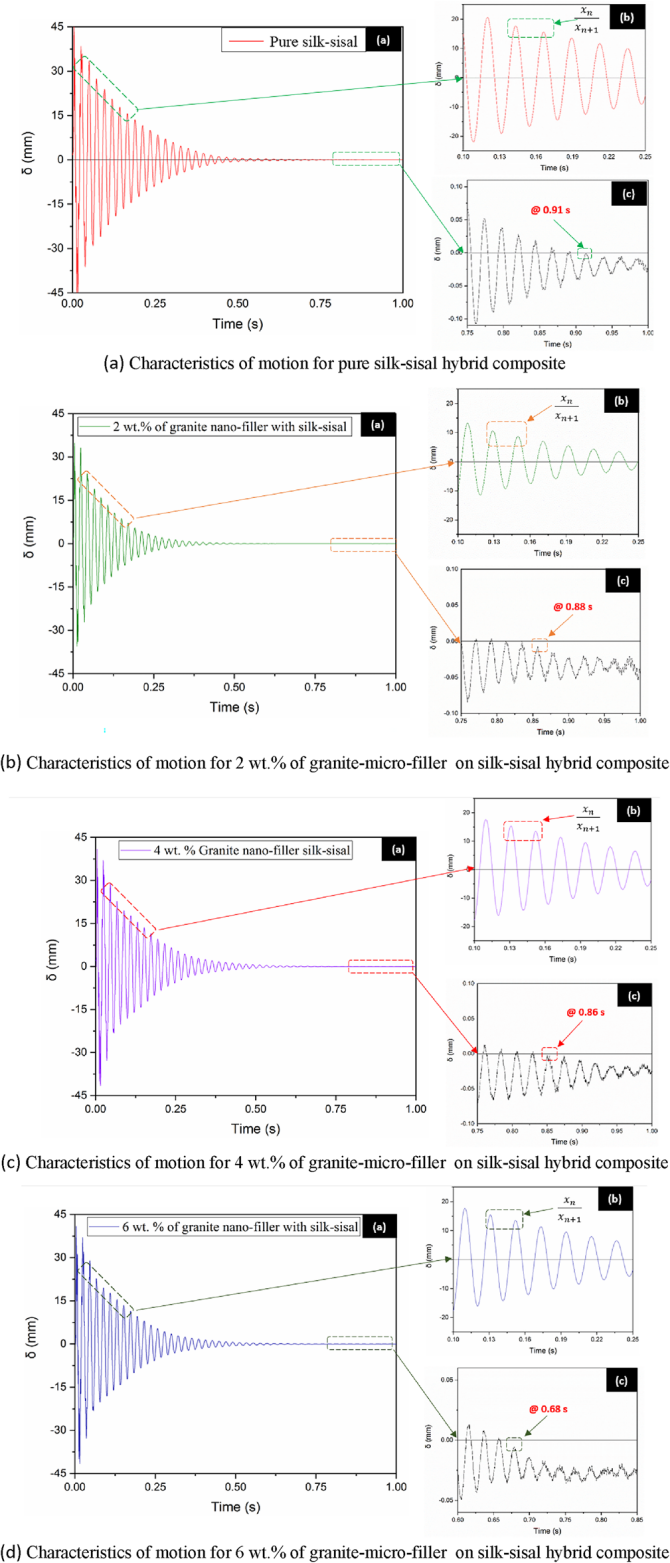
Plot (**a**) Experimental data standardized to peak with time series, (**b**) selected successive peaks for damping factor, (**c**) time at which frequency gets zero or negative.

In 2019, Jared A. Little adopt the logarithmic decrement method to estimate the damping ratio. They infer that for better estimation of damping may be a selection of large values of “n” gives analytical findings accurate average logarithmic decrement for the period^9^. The experimental test was conducted by giving an initial displacement and the consequential response was recorded, details are shown in Fig. 5a. Highlighted in Fig. 5b was the selected peak with a constant time bound for all the samples based on the above-said equation^13^. The damping ratio was calculated from the selected two successive peaks and also to reduce the uncertainty, the average value was obtained from the three trails, details are tabulated in Table 2. Figure 5c indicated the time at which frequency gets zero or negative region.

The results from Table 2 indicated that increasing wt% of the granite-micro-filler on the silk–sisal hybrid composite increases the damping factor. This is due to granite-micro-filler properly dispersion with resin and closely packed with silk–sisal fiber mat, which leads to an increase in the damping characteristics of hybrid composite. In addition to that, granite itself has inherent properties of higher damping factor.

However, a higher damping factor was obtained by adding 6 wt% of granite-micro-filler content, which is approximately 1.77 times higher than pure silk–sisal (without Micro-filler). Similarly, 1.09 times and 1.29 times higher damping values are obtained for 2 wt% and 4 wt% of granite-micro-filler content respectively. Therefore, 6 wt% of granite-micro-filler content was found to be a more significant effect on damping characteristics.

## Microstructure and fracture morphology

### Microstructure analysis

Boding characteristic of matrix/fiber mat/granite-micro-filler are the fundamental microstructural quantities and correlates with numerous properties, such as tensile strength, flexural strength, impact strength, damping behavior, fracture, etc. This morphology study mainly focuses to characterize the variation in granite-micro-filler on silk–sisal fiber, which influences the mechanical, damping, and thermal behaviors.

**Figure 6:**
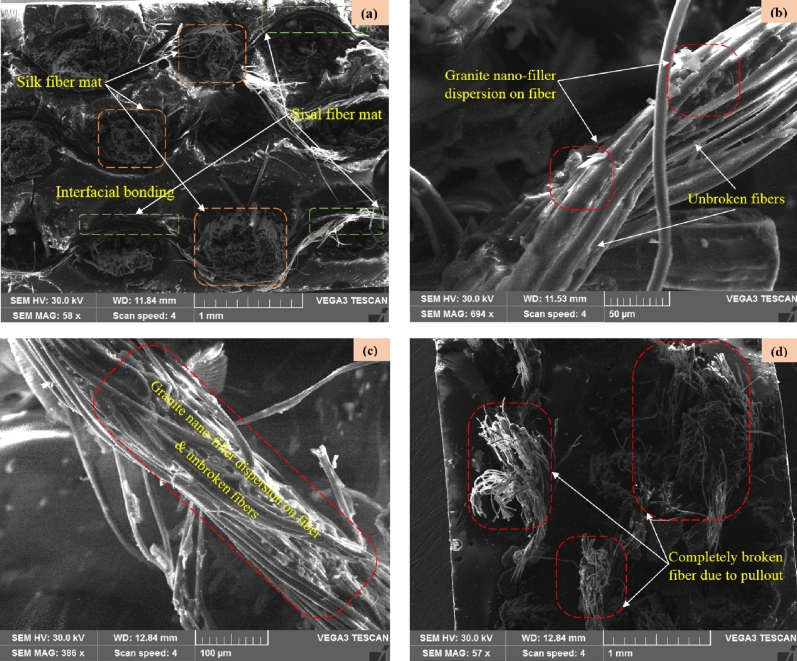
Microstructure images of (**a**) weave of silk–sisal fiber mate, and (**b**–**d**) characteristics and granite-micro-filler inclusiveness of fiber mat.

The microstructure image of the developed silk–sisal hybrid composite was shown in Fig. 6a–d, which was composed of different regions of the plain weave of fiber mat, and characteristics under the various test responses. The woven structure of the silk–sisal fiber mat is shown in the micrographs image in Fig. 6a, which consists of a plain weave with two (longitudinal and transverse) directions, the green color indicates the sisal fiber and the orange color shows the silk fiber, with closely packed matrix/fiber mat/granite-micro-filler s. i.e. the well-ordered and closed weave structure of silk–sisal was evident to possess higher mechanical properties^19^, compare to pure silk and pure sisal composite^26^. Similarly, Fig. 6b and c shows the uniform dispersion of granite nan-filler on fibers (Fig. 6b on longitudinal and Fig. 6c on transverse directions). However, the matrix/fiber/granite-micro-filler is properly bonded, because of that perfect volume fraction. However, Fig. 6d shows the broken fiber due to pulling out, i.e., during pullout, the majority of the load was on the vertically oriented fiber rather than the horizontally oriented fiber. Thus, microstructure analysis strongly evident that granite-micro-filler was dispersed on silk–sisal fiber mat and matrix are closely packed.

### Fracture morphology analysis

The interfacial characteristic and the compatibilities among fiber mats (weave silk–sisal)/resin/granite-Micro-filler) are discussed in the previous section. The mechanical responses of tensile, flexural, and impact strength of pure silk–sisal and different wt% of granite-micro-filler compositions studied and compared with existing research outcomes on sisal-banana, details are tabulated in Tables 1 and 2. Tables indicated that 1.3, 1.15, 1.8, and 1.35 times improved tensile than sisal-banana composition. It is well evident that silk–sisal has given perfect composition to replace the sisal-banana. Figure 6d illustrates the failure mechanism of the tensile strength samples, ordination in longitudinal only broken completely under the pullout condition. To summarize the tensile, a clear trend was observed for silk–sisal composition to increase the tensile strength than sisal-banana. All granite nan-filler content compositions also displayed the highest tensile breaking energy. Similarly, 1.59, 1.75, 1.98, and 1.73 times higher flexural strength and 0.43, 0.79, 0.41, and 0.79 times lesser impact strength were obtained. Based on the damping, and mechanical responses of pure silk–sisal and 6 wt% of granite-micro-filler compositions further taken to discuss the fracture morphology analysis.

**Figure 7:**
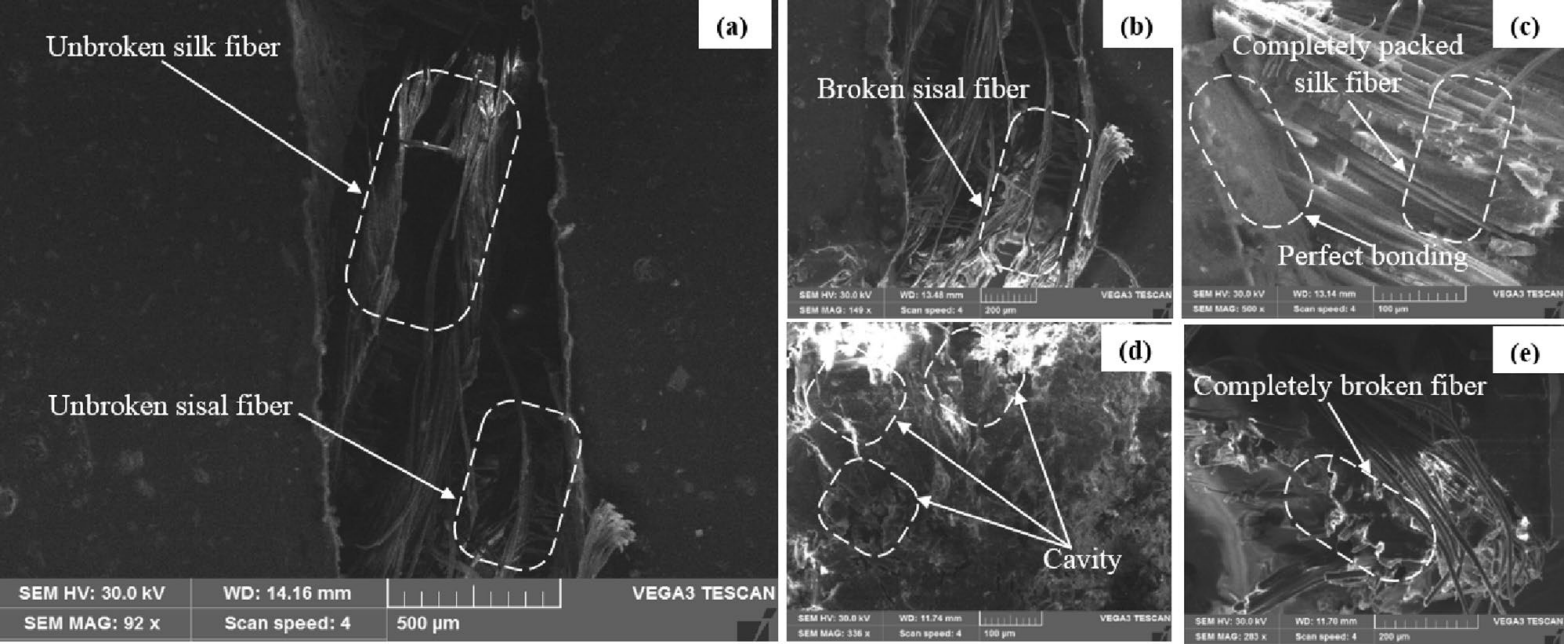
Flexural fracture microstructure images of pure silk–sisal (**a**) side view, and (**b**–**d**) characteristics of magnified view of different region.

**Figure 8:**
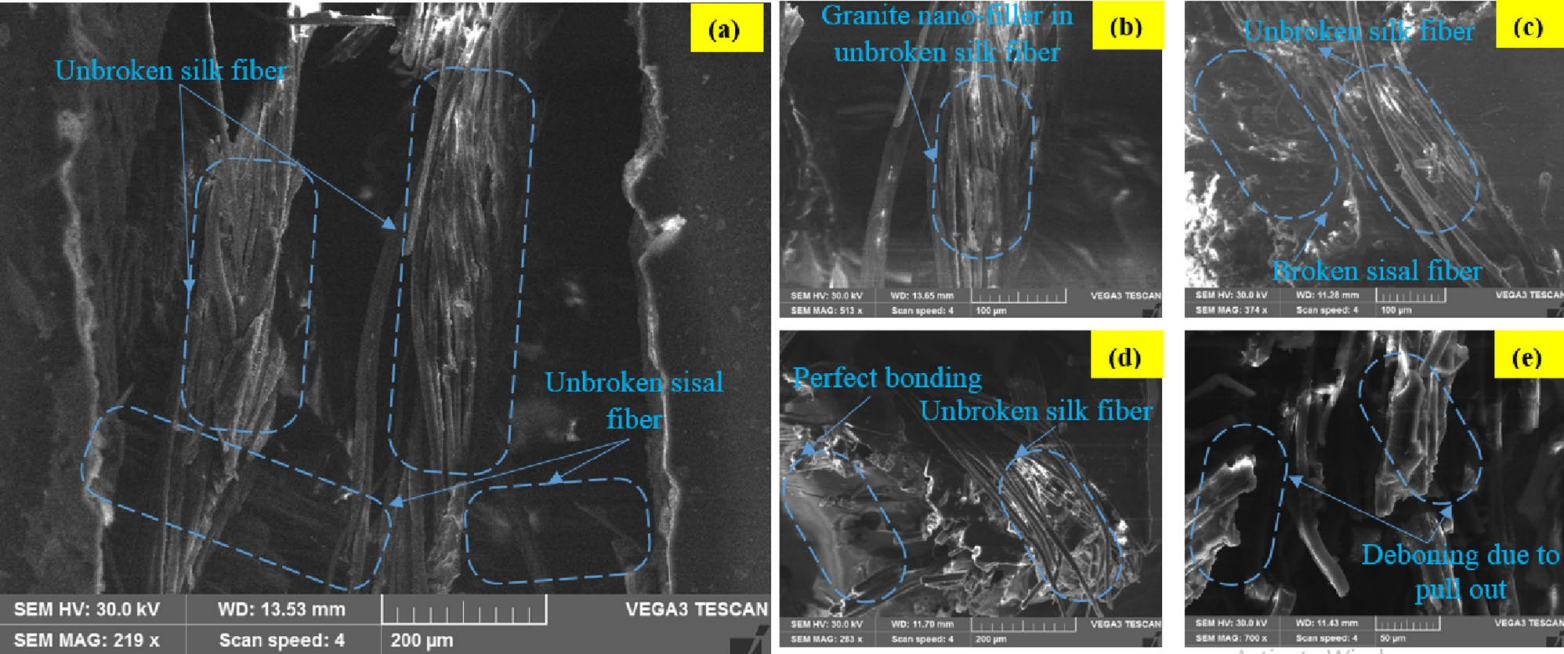
Flexural fracture microstructure images of 6 wt% of granite-micro-filler on silk–sisal (**a**) side view, and (**b**–**d**) characteristics of a magnified view of different region.

The study aims to replace the sisal-banana for the automobile application, with existing research outcomes flexural strength is one of the important factions to desire the functionality of any materials^27^. In that flexural fractography images (pure silk–sisal and 6 wt% of granite-Micro-filler) are considered for further analysis, details in Figs. 7 and 8.

Most of the researchers studied interfacial and the compatibility behavior between sisal/ banana and epoxy resin^27^ and^13^. They reported better interfacial bonding in silk-epoxy/silk-epoxy composites than in banana-epoxy composites. Generally, silk and sisal have better adhesive characteristics with epoxy, the composition of silk–sisal gives good mechanical characteristics evidenced in Tables 1 and 2. However, cracks propagation under the loading roller could be associated with the flexural failure of pure silk–sisal and 6 wt% of granite-Micro-filler, as shown in Figs. 7 and 8 with associated figures. Silk–sisal compositions are observed, possessing higher bending deformation and ductile failure nature. In both compositions unbroken fibers are displayed in Figs. 7a and 8a. in addition, Fig. 7b–e inferred that some regions are found that broken silk fiber and voids, whereas perfect boding and unbroken fiber were observed in Fig. 8b–e. This is due to the contribution of granite-Micro-filler, which is strongly evident in Table 3.

**Table 3:** Properties of silk, banana, and sisal fibers.

Fiber	% of weight gain at ${t}_{\infty }$	S	D (mm^2^/s)	P (mm^2^/s)
Pure silk–sisal	2.35	1.024	0.092	0.094
2 wt% of granite-micro-filler	1.89	1.019	0.069	0.071
4 wt% of granite-micro-filler	2.19	1.022	0.115	0.118
6 wt% of granite-micro-filler	1.51	10.12	0.119	0.121

*S*, Sorption coefficient; D, Diffusion coefficient; P, Permeability coefficient.

## Physical properties of silk–sisal hybrid composite

### Water absorption characteristics

**Figure 9:**
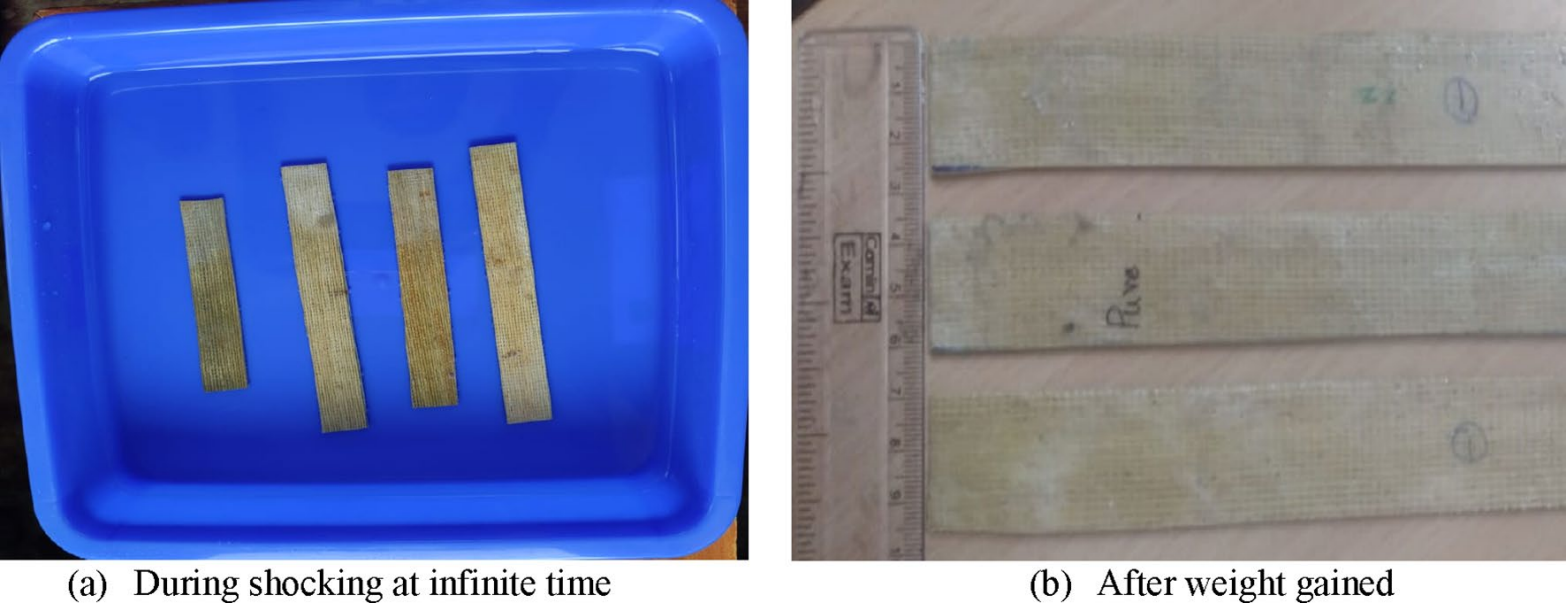
Typical water absorption samples.

The main objective of the study was to determine the relative water absorption rate by pure silk–sisal and different wt% of granite-micro-filler on silk–sisal composition as a function of time by measuring the specimen mass^27^. The silk–sisal hybrid composite samples were prepared and tested as per the ASTM 570 standard under the room temperature condition^27^ and ensured curved corners to avoid non-uniformity of water diffusion^17^, details in Fig. 9. Hot oven was used to dry all the samples for a day at 50 °C and then cooled to atmospheric temperature. The drying process was repeated until the weight of the samples remains constant and recorded the weight using a digital scale. Finally, the samples were immersed in a water bath at different times at atmospheric temperatures^20^. In this study, a constant time (0 h., 24 h., 48 h., 72 h., and 96 h.,) delay was used to measure the weight gain of all the samples. At each interval of time, all samples were removed from the water and were weighed using a digital scale immediately after they were dried with a dry cloth. The amount of water absorbed by the composites (in percentage) was calculated using Fickian as well as non-Fickian characters, which in Eq. (3)

3 $${M}_{\left(r\right)}\text{t}=\frac{{W}_{t}-{W}_{0}}{{W}_{0}}$$

where M(r) t is the relative water absorption of the specimen, W_t_ is the specimen weight at each time interval and Wo is the initial water specimen respectively.

In addition, diffusion coefficient (D), is calculated to address the solvent ability in molecules to move among the polymer region^6^, which can able to calculate in Eq. 4.

4 $$D= \pi \left(\frac{\theta h}{{4Q}_{\infty }}\right)$$

where $\theta$ is the slope of the linear portion of the sorption curve and h is the initial sample thickness in mm.

Also, the water molecule’s permeability through the developed hybrid composite is based on the sorption of water by the fiber. For that, Eq. 5 has been used to calculate the sorption coefficient (S), which is related to the equilibrium of the principal penetrated.

5 $$S=\frac{{Q}_{\infty }}{{Q}_{t}}$$

where ${Q}_{\infty }$ and ${Q}_{t}$ are the percentages of a molar of water absorption at the time ${t}_{\infty }$ and at time t. The permeability coefficient (P) is calculated with given Eq. 6, which infers the net effect of diffusion, and sorption.

6 $$P=DxS$$

**Figure 10:**
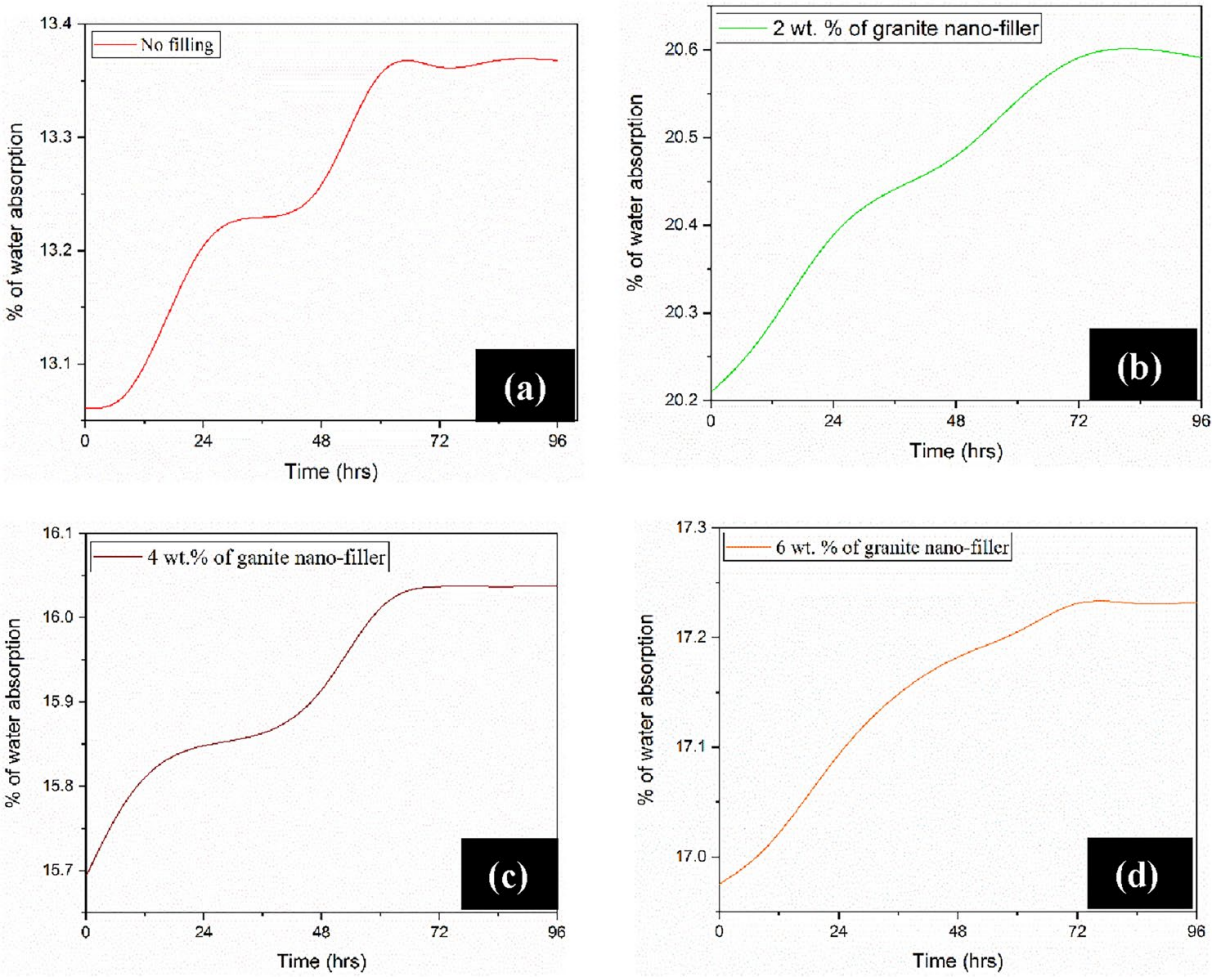
Water absorption characteristics of developed silk–sisal hybrid composites.

Kinetics of water absorption was plotted against the time, detailed in Fig. 10, and observed that all the compositions of samples were saturated from the range of 50–68 h. Experimental results inferred that, 6 wt% of granite-micro-filler samples has a lower water absorption and permeability coefficient than remaining compositions including pure silk–sisal.

### Biodegradability

**Figure 11:**
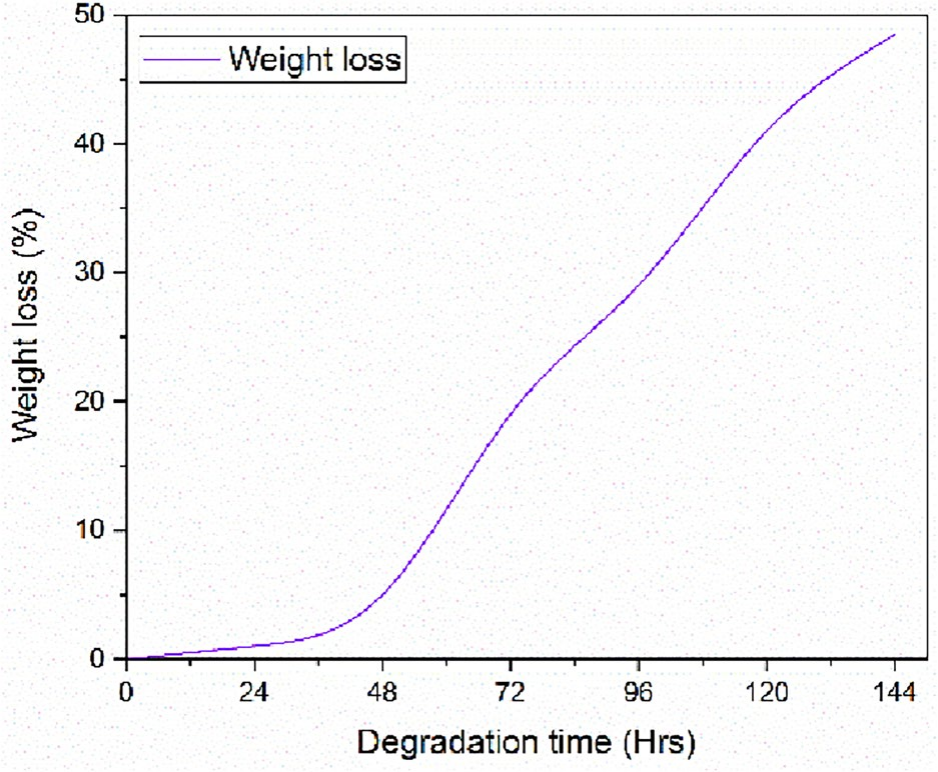
Weight reduction in the percentage.

The samples were buried in the soil for 144 h. and the changes in weight and morphology of films were observed continuously. It was found that the percentage of weight loss of each silk–sisal fiber (with and without Micro-filler) was continuously increased when the soil burial time was increased which indicated the biodegradable properties of fiber, detailed in Fig. 11.

During degradation, gelatin is attacked by microorganisms present in soil and through the cutting edge surfaces. The strength loss rate of silk–sisal composite depended on the content of the antibacterial compound. Before the biodegradation test, the surface of all samples was smooth. The samples began to degrade after 144 h. of soil burial test as observed from the roughness and the hole formation on the film surface which were due to the enzymatic activity of the microorganisms in soils. In addition to that, invertebrates and insects also be consuming the silk–sisal hybrid composite as food. Mechanical damage is also presented due to gnawing activities of termites, insects, and rodents.

## Conclusion

In this paper, the effect of different wt% of granite-micro-filler on silk–sisal hybrid composite is studied. The mechanical properties, free damping behavior, and physical properties of pure silk–sisal and different wt% of granite-micro-filler on silk–sisal hybrid composite were examined by standard experimental methods. The following conclusions are as:

- The tensile, flexural, and impact strength of the developed silk–sisal hybrid composite was increased with the increase in wt% of granite-micro-filler content than pure silk–sisal. Which evident that granite-micro-filler has a significant effect to improve the mechanical properties of silk–sisal. Approximately 5% of improved tensile strength value was obtained by adding 6 wt% granite-micro-filler on silk–sisal. Similarly, flexural strength value also improved 9% for the same wt% of granite-Micro-filler.
- Additionally, the tensile and flexural fracture morphology analysis inferred that composed plain weave of silk–sisal fiber mat with two (longitudinal and transverse) directions possesses higher mechanical properties, due to granite-micro-filler dispersed on silk–sisal fiber mat and resin with closely packed.
- Furthermore, there is no acceptable change in impact behavior by adding granite-micro-filler on silk–sisal, it possesses equal response to pure silk–sisal hybrid composite. This is due to the higher stiffness value of silk–sisal composition could decrease the impact strength and also restrain shearing behavior on fibers mat.
- The improved damping factor was obtained by adding wt% of granite-micro-filler on the silk–sisal hybrid composite. This could be granite-micro-filler perfectly dispersion with resin and closely packed with silk–sisal fiber mat and also granite itself having inherent properties of higher damping factor.
- The higher damping factor was obtained by adding 6 wt% of granite-micro-filler content, which is approximately 75.8% higher than pure silk–sisal. Similarly, 9% and 30% for 2 wt% and 4 wt% of granite-micro-filler content respectively. Therefore, 6 wt% of granite-micro-filler content was found to be a more significant effect on the silk–sisal hybrid composite structure.
- The physical properties of the silk–sisal hybrid composite were studied by water absorption and biodegradable tests. Results reveal that, 6 wt% of granite-micro-filler samples absorbed less water at an infinite time and low coefficient of permeability than remaining compositions including pure silk–sisal. In addition, acceptable degradation was found during the biodegradability test.
- Therefore, a developed silk–sisal hybrid composite added with granite-micro-filler is one of the better alternatives to enhance the damping characteristics.

## Data Availability

Influence of granite nanoparticles on the mechanical, damping and antimicrobial properties of silk-sisal hybrid nanocomposites. All data generated or analysed during this study are included in this published article and there is no separate file for dataset.
